# 

**Published:** 1892-05

**Authors:** 


### CONTENTS—MAY, 1892.—VOL. VII, No. 11.

ORIGINAL ARTICLES—

Conservative Laparotomy; by Prof. K. F. Slawiansky, St. Petersburg. Translated from the Russian for this Journal ........ 386

SOCIETY NOTES—

Synopsis of Proceedings Twenty-third Annual Meeting of the Texas State Medical Association at Tyler 389

Notes of the Tyler Meeting T. S. M. A ........ 396

EDITORIAL—

Retrench or Bankrupt—Comparative Cost of the Transactions Each Year ........ 399

Medical Department University of Texas ........ 402

Sat Down Upon ........ 407

Biographical Sketch of Dr. Osborn ........ 408

On Corns and Other Things ........ 409

Rousing Homeopathic Demonstration ........ 410

MEDICAL NEWS AND MISCELLANY ........ 411

BOOK NOTICES AND REVIEWS ........ 417

PUBLISHER'S NOTES ........ 420

